# Supplementary material for: Hybrid Models and Biological Model Reduction with PyDSTool
Source: PLoS Comput Biol. 2012 Aug 9;8(8):e1002628. doi: 10.1371/journal.pcbi.1002628 (PMC3415397; doi:10.1371/journal.pcbi.1002628)
Supplement: Text S4 — Complete source code for the PyDSTool package (version 0.88.120504). Includes API documentation and help files linking to web pages. This file is identical to the current public release on Sourceforge.net. (ZIP) [file pcbi.1002628.s004.zip › PyDSTool/html/PyDSTool.PyCont.Plotting.pargs-class.html]

xml version="1.0" encoding="ascii"?


PyDSTool.PyCont.Plotting.pargs


| Home | Trees | Indices | Help | PyDSTool |
| --- | --- | --- | --- | --- |

Package PyDSTool ::
Package PyCont ::
Module Plotting ::
Class pargs

[hide private]

[frames] | no frames]

# Class pargs

source code

```
 object --+    
          |    
common.args --+
              |
             pargs
```

---


|  |  |
| --- | --- |
| Instance Methods | [hide private] |

|  |  |
| --- | --- |
| \_\_getitem\_\_(self, key) | source code |

|  |  |
| --- | --- |
| \_\_repr\_\_(self, ct=0, res=None)  str(x) | source code |

|  |  |
| --- | --- |
| \_\_str\_\_(self, ct=0, res=None)  str(x) | source code |

|  |  |
| --- | --- |
| fromLabel(self, label, key=None) | source code |

|  |  |
| --- | --- |
| get(self, objtype, bylabel=None, byname=None, bytype=None, bylegend=None, obj=None, ct=0) | source code |

|  |  |
| --- | --- |
| toggleLabel(self, visible=`'``on``'`, refresh=True) | source code |

|  |  |
| --- | --- |
| toggleLabels(self, visible=`'``on``'`, bylabel=None, byname=None, bytype=None, ct=0) | source code |

|  |  |
| --- | --- |
| togglePoint(self, visible=`'``on``'`, refresh=True) | source code |

|  |  |
| --- | --- |
| togglePoints(self, visible=`'``on``'`, bylabel=None, byname=None, bytype=None, ct=0) | source code |

|  |  |
| --- | --- |
| toggleCurve(self, visible=`'``on``'`, refresh=True) | source code |

|  |  |
| --- | --- |
| toggleCurves(self, visible=`'``on``'`, bylegend=None, byname=None, bytype=None, ct=0) | source code |

|  |  |
| --- | --- |
| toggleCycle(self, visible=`'``on``'`, refresh=True) | source code |

|  |  |
| --- | --- |
| toggleCycles(self, visible=`'``on``'`, bylegend=None, byname=None, bytype=None, ct=0) | source code |

|  |  |
| --- | --- |
| toggleAll(self, visible=`'``on``'`, bylabel=None, byname=None, bytype=None, ct=0) | source code |

|  |  |
| --- | --- |
| setLabel(self, label, refresh=True) | source code |

|  |  |
| --- | --- |
| setLabels(self, label, bylabel=None, byname=None, bytype=None, ct=0) | source code |

|  |  |
| --- | --- |
| setLegend(self, legend, refresh=True) | source code |

|  |  |
| --- | --- |
| setLegends(self, legend, bylegend=None, byname=None, bytype=None, ct=0) | source code |

|  |  |
| --- | --- |
| refresh(self) | source code |

|  |  |
| --- | --- |
| clean(self)  Cleans plotting structure (e.g. | source code |

|  |  |
| --- | --- |
| clear(self, refresh=True) | source code |

|  |  |
| --- | --- |
| clearall(self) | source code |

|  |  |
| --- | --- |
| delete(self, refresh=True) | source code |

|  |  |
| --- | --- |
| deleteall(self) | source code |

**Inherited from `common.args`**:
`__add__`,
`__cmp__`,
`__contains__`,
`__delitem__`,
`__eq__`,
`__ge__`,
`__gt__`,
`__init__`,
`__iter__`,
`__le__`,
`__len__`,
`__lt__`,
`__ne__`,
`__setitem__`,
`copy`,
`fromkeys`,
`has_key`,
`info`,
`items`,
`iteritems`,
`iterkeys`,
`itervalues`,
`keys`,
`pop`,
`popitem`,
`setdefault`,
`update`,
`values`

**Inherited from `common.args`** (private):
`_infostr`

**Inherited from `object`**:
`__delattr__`,
`__getattribute__`,
`__hash__`,
`__new__`,
`__reduce__`,
`__reduce_ex__`,
`__setattr__`


|  |  |
| --- | --- |
| Properties | [hide private] |

**Inherited from `object`**:
`__class__`


|  |  |
| --- | --- |
| Method Details | [hide private] |

|  |  |
| --- | --- |
| \_\_getitem\_\_(self, key)  *(Indexing operator)* | source code |

Overrides: common.args.\_\_getitem\_\_

|  |  |
| --- | --- |
| \_\_repr\_\_(self, ct=0, res=None)  *(Representation operator)* | source code |

str(x)

Overrides: object.\_\_repr\_\_: *(inherited documentation)*

|  |  |
| --- | --- |
| \_\_str\_\_(self, ct=0, res=None)  *(Informal representation operator)* | source code |

str(x)

Overrides: object.\_\_str\_\_: *(inherited documentation)*

|  |  |
| --- | --- |
| get(self, objtype, bylabel=None, byname=None, bytype=None, bylegend=None, obj=None, ct=0) | source code |

Overrides: common.args.get

|  |  |
| --- | --- |
| clean(self) | source code |

Cleans plotting structure (e.g. deleted) Also gets rid of nonexisting
figures if they've been deleted by an outside source. This method is a
little clumsy since it has to spawn a dummy plot if a plot has been
deleted, but who cares, it works.

|  |  |
| --- | --- |
| clear(self, refresh=True) | source code |

Overrides: common.args.clear

  


| Home | Trees | Indices | Help | PyDSTool |
| --- | --- | --- | --- | --- |

|  |  |
| --- | --- |
| Generated by Epydoc 3.0.1 on Fri May 4 15:24:07 2012 | http://epydoc.sourceforge.net |
